# Case report: Pseudoprogression mimicking neoplastic recurrence three months after completion of proton beam therapy for an IDH-mutant astrocytoma CNS WHO grade 3

**DOI:** 10.3389/fonc.2025.1397912

**Published:** 2025-01-30

**Authors:** Liv Cathrine Heggebø, Ida Maria Henriksen Borgen, Hanne Blakstad, Cathrine Saxhaug, Pål André Rønning, Pitt Frederik Niehusmann, Katja Werlenius, Malin Blomstrand, Petter Brandal

**Affiliations:** ^1^ Department of Oncology, Oslo University Hospital, Oslo, Norway; ^2^ Institute of Clinical Medicine, University of Oslo, Oslo, Norway; ^3^ Department of Physical Medicine and Rehabilitation, Oslo University Hospital, Oslo, Norway; ^4^ Department of Radiology, Oslo University Hospital, Oslo, Norway; ^5^ Department of Neurosurgery, Oslo University Hospital, Oslo, Norway; ^6^ Department of Pathology, Oslo University Hospital, Oslo, Norway; ^7^ Department of Oncology, Sahlgrenska University Hospital, Gothenburg, Sweden; ^8^ The Skandion Clinic, Uppsala, Sweden; ^9^ Institute for Cancer Genetics and Informatics, Oslo University Hospital, Oslo, Norway

**Keywords:** IDH-mutant astrocytoma, diffuse glioma, pseudoprogression, proton beam therapy, radiotherapy

## Abstract

**Background:**

Radiation-induced changes following proton beam therapy in isocitrate dehydrogenase (*IDH*)-mutated diffuse central nervous system (CNS) World Health Organization (WHO) grade 2 and 3 gliomas are not well characterized. We present a patient with an *IDH*-mutant astrocytoma CNS WHO grade 3 treated with proton beam therapy and with postradiation MRI changes suggestive of neoplastic progression that surprisingly turned out to be reactive.

**Case presentation:**

A man in his twenties underwent surgery with a near gross total resection for what turned out to be an *IDH*-mutant astrocytoma CNS WHO grade 3. He was included in the PRO-GLIO trial and randomized to receive proton beam therapy to a total dose of 59.4 Gray (Gy) relative biological effectiveness (RBE). Four weeks after completion of radiotherapy, adjuvant temozolomide was commenced. All treatment was well tolerated, and the patient was in excellent general condition. Surprisingly, magnetic resonance imaging (MRI) examination three months after completion of radiotherapy showed what was highly suggestive of a distant recurrence. The patient underwent resective surgery about seven months after his first surgery. Histological examination showed inflammatory changes without neoplastic tissue, albeit not very typical for postradiation changes. Adjuvant chemotherapy with temozolomide was continued.

**Conclusion:**

The presented case clearly shows that caution must be taken when interpreting cerebral MRI changes postradiation, and in particular after proton therapy. Further understanding of this subject is crucial to distinguish between patients requiring intensified antineoplastic treatment and those for whom maintaining current therapy or ongoing watchful waiting is advisable.

## 1 Introduction

Isocitrate dehydrogenase (*IDH)-*mutant astrocytoma central nervous system (CNS) World Health Organization (WHO) grade 3 is a subtype of adult type diffuse gliomas and is, in principle, an incurable disease. However, median estimated survival for affected individuals is about eight years with considerable inter-individual differences (1, 2). Treatment therefore needs to be delicately balanced, focusing on tumor control as well as an optimal quality of life. Standard therapy for patients with *IDH-*mutated diffuse astrocytoma CNS WHO grade 3 consists of maximally safe surgical resection followed by radiotherapy to a total dose of 59.4 Gray (Gy) and 12 adjuvant courses of temozolomide (3–5). All these anti-neoplastic therapies are prone to side effects, including radiotherapy, which is known to carry a risk of detrimental late effects (6–11). Proton therapy is an increasingly used radiotherapeutic modality with physical properties that facilitate improved preservation of healthy tissue compared to photon therapy (12–16). This is an appealing quality for patients with relatively favorable lifetime expectancies, such as diffuse gliomas CNS WHO grade 2 and 3. However, their diffuse infiltrative nature poses a potential hazard. The ongoing PRO-GLIO study investigates whether proton therapy is safe and beneficial for *IDH-*mutated diffuse gliomas grade 2 and 3 (17), thereby seeking to establish whether proton therapy should be implemented as standard of care for this patient group (18, 19).

With new treatment modalities, new clinical conundrums appear. Pseudoprogression is a phenomenon most often seen in high-grade gliomas following photon radiotherapy (20–25). It may, however, also appear after radiotherapy for diffuse grade 2 and 3 gliomas (22, 26–28). Pseudoprogression imaging characteristics, timing related to radiotherapy, susceptible locations, and incidence following proton radiotherapy might be different than after photon therapy, partly related to protons’ slightly higher relative biological effectiveness (RBE) (29–32). The most critical clinical task is distinguishing pseudoprogression from neoplastic progression, an endeavor that is often challenging and lacks universally accepted guidelines or criteria.

In the PRO-GLIO trial, a previously healthy young man diagnosed with an *IDH-*mutant astrocytoma CNS WHO grade 3 was randomized to protons given to the total dose of 59.4 Gy. Three months after the completion of radiotherapy, magnetic resonance imaging (MRI) showed what was highly suspicious of a distant recurrence. The patient was in excellent health with no new symptoms, and the new lesion was treated surgically as the appearance and location did not suggest pseudoprogression. This case highlights the need to include pseudoprogression as a differential diagnosis whenever new postradiation lesions appear.

## 2 Case presentation and diagnostics assessment

**Figure 1:**
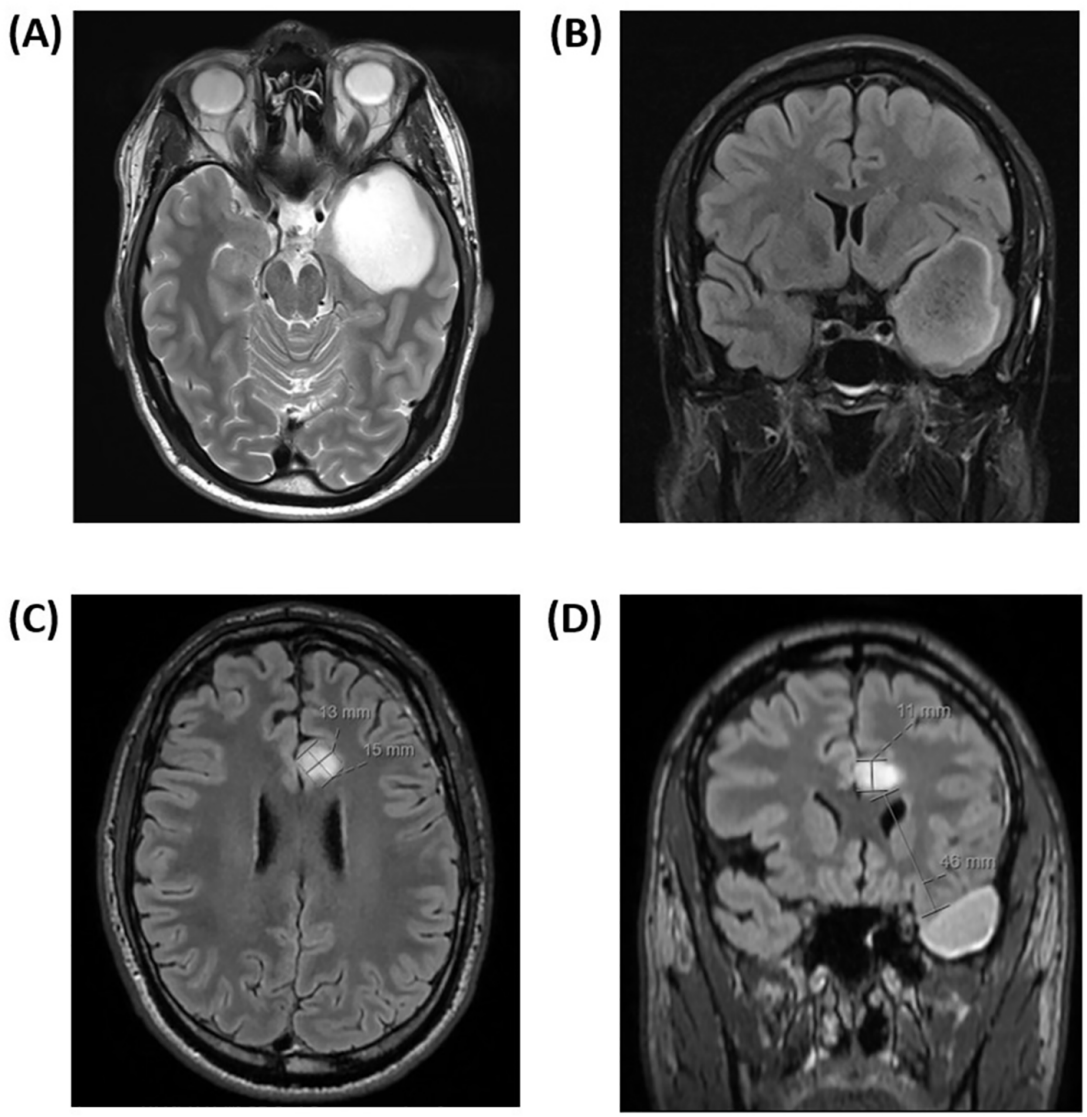
**(A, B)** Axial magnetic resonance contrast-enhanced T2/FLAIR image sequence prior to the first tumor resection showed a nonenhancing cystic lesion in the left temporal lobe, suspicious for diffuse low-grade glioma. The tumor measured 5 x 4 x 5 centimeters in its largest dimension. **(C, D)** Axial magnetic resonance contrast-enhanced T2/FLAIR image sequence three months after completion of proton beam therapy. A new 15 x 13 x 11 millimeters nonenhancing lesion, highly suspicious of distant neoplastic recurrence in the left subcortical, parasagittal left limbic area was found. The appearance of the lesion was relatively well-defined and expansive, and it was located 46 millimeters from the resection cavity.

A young man in his twenties presented with epileptic seizures. He was otherwise healthy and had no prior medication. An electroencephalogram (EEG) conducted after a hyperventilation episode detected focal pathological activity in the left frontotemporal region, and a sleep-deprived EEG gave rise to suspicion of a structural abnormality in the same region. A nonenhancing lesion measuring 5 x 4 x 5 centimeters (cm), suspicious for a diffuse low-grade glioma, was identified in the left temporal lobe by MRI (**Figures 1A, B**). A few weeks later, the patient underwent surgery with awake craniotomy and the use of intraoperative MRI. Only a small residual neoplastic lesion in the left insular region identified on postoperative MRI remained. The patient’s preoperative Neurological Assessment in Neuro-Oncology (NANO; 33) score was 0, whereas a mild and transient postoperative expressive aphasia led to a NANO score of 0-1. He was in good general condition with Karnofsky Performance Status (KPS) score of 100 before and after surgery. Examination of the tissue specimen revealed an *IDH-*mutant astrocytoma CNS WHO grade 3 with an O-6 methylguanine-DNA methyltransferase (MGMT) promotor methylation level of 5.5%.

Following resection, the patient was included in the PRO-GLIO trial and randomized to receive proton therapy, which commenced about two months following resection. Fractionation was 1.8 Gray (Gy) RBE x 33 to a total dose of 59.4 Gy RBE. Radiotherapy was delivered with two plans. The first plan was used for 23 of 33 fractions and a second plan contributed with the last 10 fractions. Replanning was done due to swelling of the skin in the patient’s left temporal region. In sum, radiation doses were within OAR tolerance doses according to the European Particle Therapy Network (EPTN) consensus (34) and standard clinical practice at the proton institution. Both plans used a 3-field technique with identical field angles; one field with a 320 gantry degree and a 90 couch degree, a second with a 70 gantry degree and a couch degree of 10, and a third with a gantry degree of 100 and a couch degree of 0. Monitor units (MU) per fraction for the first plan was 207.9, 256.2, and 249.8 for the three fields, and for the second plan 272.9, 293.4, and 289.0, respectively. Radiotherapy was well tolerated, with only mild fatigue at the end of treatment.

**Figure 2:**
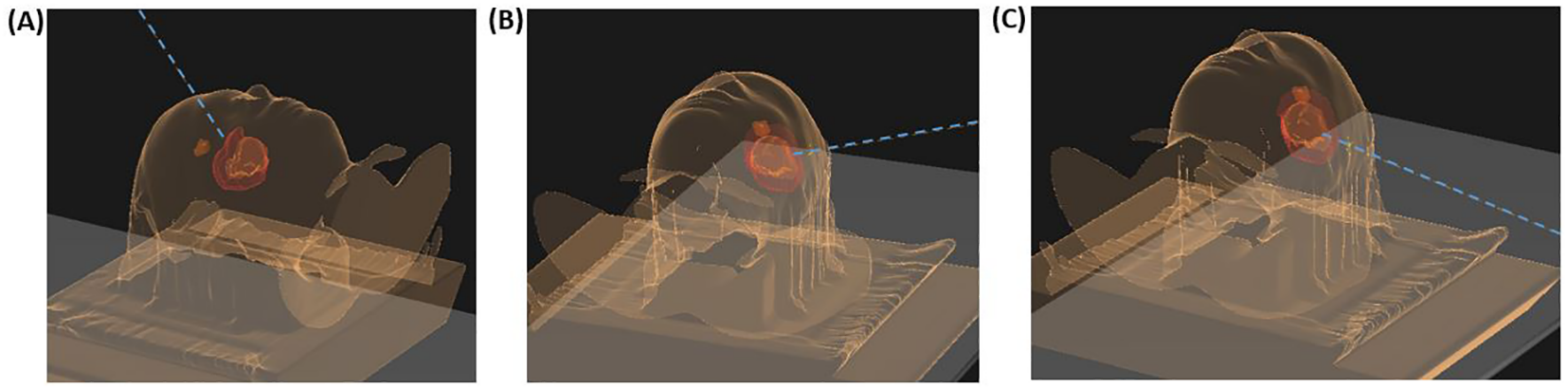
Beam angles for the patient’s proton plan in relation to the original gross target volume (GTV, orange), clinical target volume (CTV, red), and the location of the new lesion suspicious of a recurrence (brown). **(A)** displays the field with 320 gantry degree and a 90 couch degree, **(B)** the second field with a 70 gantry degree and a couch degree of 10, and **(C)** the third beam with a gantry degree of 100 and a couch degree of 0.

One month after completion of radiotherapy, standard adjuvant chemotherapy with temozolomide was initiated and well tolerated. The first postradiation MRI undertaken three months after completion of radiotherapy showed stable disease in the primary tumor area; however, surprisingly, with a new nonenhancing lesion in the left anterior cingulate gyrus measuring 15 x 13 x 11 millimeters (mm; **Figures 1C, D**). The lesion appeared well-defined and expansive, with a high T2/FLAIR (Fluid-Attenuated Inversion Recovery) signal. Most of the lesion exhibited high diffusion (1.5 x 10^-3^ mm^2^/s) with a peripheral rim of low diffusion (0.9 x 10^-3^ mm^2^/s). The T2/FLAIR mismatch present in the primary lesion was not observed in this case. Apart from this, the new lesion appeared highly suspicious for a distant recurrence of the *IDH*-mutant astrocytoma. **Figure 2** illustrates the radiotherapy beam angles in relation to the original GTV and CTV, as well as the location of the new lesion which was radiologically deemed suspicious of a recurrence. The patient was in excellent general condition with KPS score 100 without new symptoms. At this time point, he had received only two courses of temozolomide, using ondansetron as an anti-emetic only on the five treatment days. Apart from this, the only medication he used was an anti-epileptic (levetiracetam 1000 milligrams two times daily). A neuropsychological assessment conducted as part of the PRO-GLIO trial did not uncover any new cognitive deficits.

**Figure 3:**
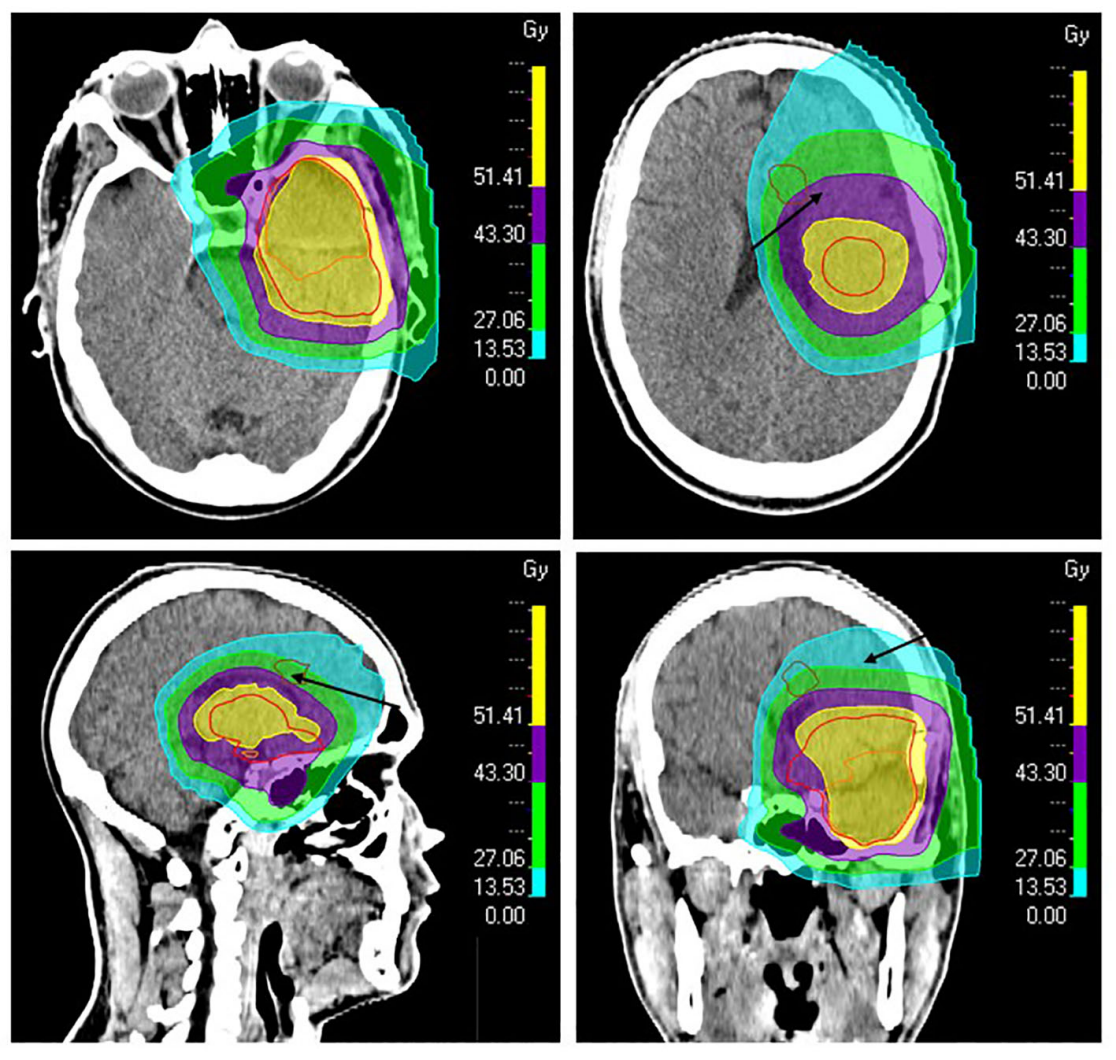
The patient’s proton plan showing isodose levels and radiotherapy target volumes. The resection cavity after primary surgery and the suspected residual tumor were delineated as gross target volume (GTV, orange). A margin of 15 mm was added to GTV to define the clinical target volume (CTV, red), which was also modified against natural anatomical barriers. The new lesion found by MRI three months after proton therapy was retrospectively delineated (brown, arrows) to calculate dose levels in the region of the lesion. The 51.4 Gray (Gy) (95%) isodose line is shown in yellow, 43.3 Gy (80%) in purple, 27.1 Gy (50%) in green, and 13.5 Gy (25%) in turquoise.

After a multidisciplinary discussion, it was decided to offer the patient a resection followed by new radiotherapy for the presumed distant neoplastic progression. A preoperative MRI was performed the day before the second resection and the lesion had increased in size compared to the MRI four weeks earlier; now measuring 21 x 14 x 8 mm and increasing the suspicion of tumor progression. The patient accepted, and a gross total resection (GTR) was achieved with a pre-and postoperative NANO score of 0. The second surgery was performed 7 months after his first resection. Surprisingly, histopathological and molecular biological examination of the tissue specimen did not reveal active neoplastic tissue. Macrophages and perivascular immune cell accumulations were seen - fitting well with inflammation, albeit not typical for postradiation changes. Retrospectively, D_98%_ (radiotherapy dose received by 98%) of the new lesion was estimated to be 15 Gy RBE (**Figure 3**); however, parts of the new lesion had received up to 50 Gy RBE. A new multidisciplinary discussion decided against offering additional radiotherapy, opting instead to continue adjuvant temozolomide.

**Figure 4:**
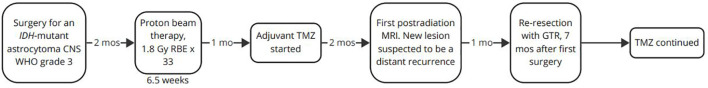
The patient’s timeline. CNS, central nervous system; GTR, gross total resection, Gy, Gray; IDH, isocitrate dehydrogenase; mo, month; mos, months; MRI, magnetic resonance imaging; RBE, relative biological effectiveness; TMZ, temozolomide; WHO, World Health Organization.

MRI two months after the second surgery showed no signs of neoplastic activity. The patient´s timeline is summarized in **Figure 4**. Fortunately, the patient has no sequelae following surgery. The last MRI taken one year following the second surgery shows no new lesions, and the patient is in excellent general condition.

## 3 Discussion

Pseudoprogression is a relatively common phenomenon following radiotherapy for brain neoplasms and is often hard to distinguish from actual neoplastic progression.

Three months following completion of proton therapy, our patient underwent resection for a new lesion that was highly suspicious for a distant neoplastic recurrence of an *IDH*-mutant astrocytoma CNS WHO grade 3. Surprisingly, examination of the tissue specimen showed no evidence of neoplasia but changes compatible with inflammation. Although not characteristic for postradiation changes, proton therapy is suspected to be the etiological basis for the lesion. Adjuvant chemotherapy with temozolomide was continued following surgery, as initially planned.

Usually, pseudoprogression for patients with lower-grade diffuse gliomas is defined as new or increased contrast-enhancement (27). Response Assessment in Neuro-Oncology (RANO) criteria propose that neoplastic progression is most likely when the majority of a new contrast-enhancing lesion is outside the radiation field (beyond the high-dose region/80% isodose line) (24, 35). However, it has also been argued that pseudoprogression can manifest as new or increased T2/FLAIR-signal hyperintensity (28, 36). As *IDH*-mutated lower grade gliomas most often are nonenhancing, increased T2/FLAIR-signal hyperintensity may mimic the primary disease more than new contrast-enhancing lesions. Pseudoprogression is often seen in the first 12 weeks following radiotherapy in glioblastomas which are per definition *IDH* wild-type, whereas onset may be later for *IDH-*mutant lower-grade gliomas, although timing is not uniform (28, 35).

Pseudoprogression is well-known following photon therapy, but is also known to occur following the less available proton therapy. Radiotherapy with protons is increasingly used for treatment of *IDH-*mutated diffuse gliomas grade 2 and 3, and understanding the appearances of pseudoprogression is therefore highly relevant. Ritterbusch et al. suggested criteria for characterizing pseudoprogression after proton beam therapy: location in the distal end of the proton beam, small lesions (<1 cm), often multifocal, and resolving without anti-neoplastic therapy (29). In a systematic review and meta-analysis by Lu et al., the incidence of pseudoprogression was 30% for adults with low-grade diffuse gliomas following proton beam therapy, compared to 18% of patients who received photon-based intensity-modulated radiotherapy (IMRT) (30). Bronk et al. failed to identify any difference between the two modalities (37).

In the study by Ritterbusch et al., mean time to development of pseudoprogression was 15 months, ranging from 7.0-27 months, and often appearing later than what is normally seen following photon therapy (29). The latter is in contradiction to the findings by Bronk et al. who found that pseudoprogression after proton beam therapy appeared earlier than with photon therapy for patients with oligodendrogliomas grade 2 and 3; the same was not observed in patients with diffuse astrocytomas (37). Others have found that pseudoprogression appeared at median 7.6 (proton) and 12 (photon) months following radiotherapy (27, 36).

Ritterbusch et al. found no association between pseudoprogression and sex, age, *IDH-*mutation, grade, MGMT promotor methylation, 1p/19q codeletion, or chemotherapy received (29). Somewhat contradictory, Dworkin et al. found an increased risk for pseudoprogression in patients with diffuse low-grade gliomas when temozolomide was given adjuvant following proton beam therapy (36). In a study by Harrabi et al., radiation-induced brain injuries for diffuse low-grade gliomas following proton beam therapy were almost exclusively seen in the distal part of the spread-out Bragg-peak. However, in this study, only contrast-enhanced lesions were considered (31). Besides at the distal part of the proton beam and within the high-dose region, new contrast-enhancing lesions are also often located in close proximity to the ventricular system (32).

In our patient, the new lesion was unifocal, measuring over 1 cm (15 x 13 x 11 mm), located 46 mm from the edge of the primary resection cavity (**Figure 1**), and most of it was located outside the high-dose region (**Figure 3**). The lesion was located close to the ventricular system, which is a predilection site for postirradiary changes, however, and more atypical - the lesion was nonenhancing with a high T2/FLAIR signal, and it appeared as early as three months after radiotherapy completion when the patient had only received two courses of temozolomide. The only medications used in addition to temozolomide were ondansetron and levetiracetam, none of which is thought to increase the risk of pseudoprogression. Most parameters suggested that the lesion was highly suspicious for a recurrence, which nonetheless turned out to be wrong.

Patients with *IDH*-mutant gliomas grade 2 and 3 are often young and have long expected survival. All therapeutic measures must be delicately balanced to avoid unnecessary side effects. Gaining better knowledge on pseudoprogression following proton beam therapy is essential to avoid superfluous and possibly harmful therapeutic measures. In the presented case, neoplastic progression was considered the most likely explanation for the new lesion, based mainly on timing, distance to the radiotherapy target volume and MRI appearance Therefore, resection was considered the most prudent approach. However, it could have been delayed or even avoided if an accurate radiological diagnosis could have been established. The case illustrates that pseudoprogression should always be considered a differential diagnosis when new lesions appear following radiotherapy of patients with *IDH*-mutant gliomas grade 2 and 3. More knowledge about radiotherapy-induced MRI changes in these patients is needed, and we hope that the PRO-GLIO trial will contribute to close this knowledge gap.

## Data Availability

The data analyzed in this study is subject to the following licenses/restrictions: Due to strict private policy data will not be made publicly available. Data is available on study center upon request. Requests to access these datasets should be directed to licahe@ous-hf.no.
